# A common tattoo chemical for energy storage: henna plant-derived naphthoquinone dimer as a green and sustainable cathode material for Li-ion batteries†

**DOI:** 10.1039/c7ra12357d

**Published:** 2018-01-04

**Authors:** Mikhail Miroshnikov, Keiko Kato, Ganguli Babu, Kizhmuri P. Divya, Leela Mohana Reddy Arava, Pulickel M. Ajayan, George John

**Affiliations:** Department of Chemistry, Center for Discovery and Innovation, The City College of New York 85 St. Nicholas Terrace New York NY 10031 USA gjohn@ccny.cuny.edu; PhD Program in Chemistry, The Graduate Center of the City University of New York 365 5^th^ Ave New York NY 10016 USA; Department of Materials Science and Nano Engineering, Rice University 6100 Main Street MS 325 Houston TX 77005 USA ajayan@rice.edu; Department of Mechanical Engineering, Wayne State University 5050 Anthony Wayne Dr. Detroit MI 48202 USA fl8799@wayne.edu

## Abstract

The burgeoning energy demands of an increasingly eco-conscious population have spurred the need for sustainable energy storage devices, and have called into question the viability of the popular lithium ion battery. A series of natural polyaromatic compounds have previously displayed the capability to bind lithium *via* polar oxygen-containing functional groups that act as redox centers in potential electrodes. Lawsone, a widely renowned dye molecule extracted from the henna leaf, can be dimerized to bislawsone to yield up to six carbonyl/hydroxyl groups for potential lithium coordination. The facile one-step dimerization and subsequent chemical lithiation of bislawsone minimizes synthetic steps and toxic reagents compared to existing systems. We therefore report lithiated bislawsone as a candidate to advance non-toxic and recyclable green battery materials. Bislawsone based electrodes displayed a specific capacity of up to 130 mA h g^−1^ at 20 mA g^−1^ currents, and voltage plateaus at 2.1–2.5 V, which are comparable to modern Li-ion battery cathodes.

## Abstract 2

The burgeoning energy demands of an increasingly eco-conscious population have spurred the need for sustainable energy storage devices, and have called into question the viability of the popular lithium ion battery.

## Graphical abstract

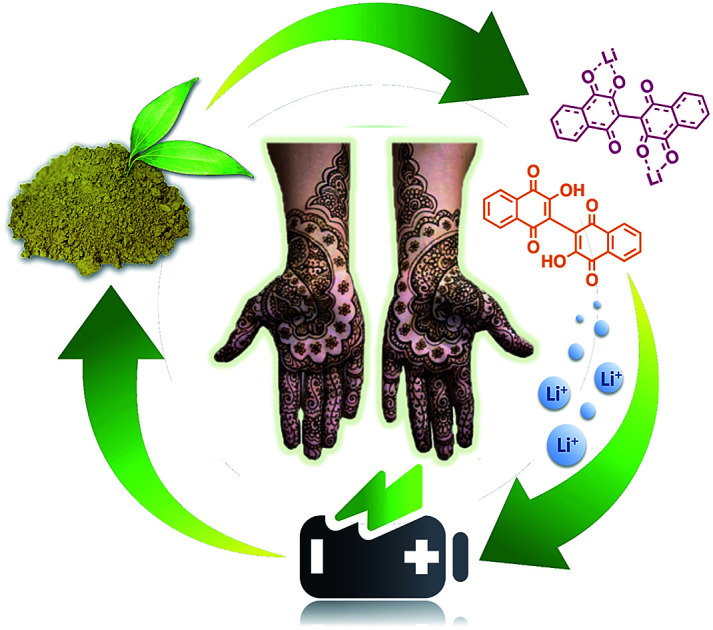

## Introduction

Lithium-ion batteries (LIBs) are a ubiquitous staple of modern electronics. However, despite their pronounced commercial achievements, LIBs have received considerable criticism due to their negative environmental impact, safety issues, and the energetic/monetary costs associated with manufacturing and recycling.^1–4^ Typical LIB cathodes are constructed of LiCoO_2_ or LiNiO_2_ cathodes,^5,6^ which require scarce and costly lithium and toxic transition metals such as cobalt.^7,8^ In addition, there is evidence to suggest that CO_2_ emissions associated with their production might outweigh the impact of their incorporation into seemingly eco-benign innovations such as electric vehicles.^3,9,10^ A number of inorganic-based materials have been proposed over the years as alternatives to LiCoO_2_. These include the less expensive and environmentally friendly LiMn_2_O_4_ and LiFePO_4,_ as well as emerging Li–O_2_ and Li–S battery technologies.^11^ However, these materials themselves suffer from a number of issues, including poor thermal and chemical stability, conductivity, and lack of compatible electrolytes.^12–15^ Groups like that of Jean-Marie Tarascon^16^ and our own^17^ were amongst the first to suggest that small organic molecules, which bind Li *via* polar oxygen-containing functional groups,^18,19^ were capable of displaying comparable electrochemical activities to more traditional electrode materials.^20,21^ Purpurin, a naturally occurring organic dye derived from the madder plant, was first shown by our group to achieve specific capacities of up to ∼150 mA h g^−1^ (comparable to LIB) as a result of binding two Li atoms.^17,21^

To reduce the carbon footprint associated with the battery lifecycle, we aim to further discover and develop easily recyclable batteries from sustainable (green) materials extracted from plants and biomass. As part of this ongoing effort, we investigated bislawsone (BL), a dimer of 2-hydroxy-1,4-naphthoquinone (lawsone). Lawsone (LS) is a pigment isolated from the leaves of the henna plant and has been utilized as a skin and hair dye since 1400 BC.^22–24^ Lawsone has been reported to readily bind sodium atoms,^25^ and has recently shown promising electrochemical activity.^26^ However, the lithium binding capacity and electrochemical activity of bislawsone as a cathode material for LIB applications has not been previously reported. Hence, we have dimerized lawsone to bislawsone (BL) through a facile one-step click chemistry reaction,^23^ which serves to increase the availability of Li-binding redox sites. We report the subsequent chemical lithiation of bislawsone to yield lithiated bislawsone (Li-BL) using mild and greener reaction conditions.^27^ Compared to existing organic electrode materials, including those derived from other electroactive quinones and organic polymers,^28–30^ our bislawsone compounds offer the advantage of being naturally sourced, and avoiding harsh or expensive catalysts/reagents. Additionally, these materials are synthesized in environmentally benign or minimally problematic solvents (water, acetonitrile, and methanol)^31^ and require minimal synthetic steps or extensive purification procedures. Functional organic electrodes constructed from Li-BL exhibited impressive reversible Li ion storage capabilities comparable to modern day inorganic cathode materials.^14,32^ This work therefore channels the often-overlooked utility of natural molecules into an eco-friendly solution for challenges facing the lithium-ion battery.

## Experimental

### Synthesis and lithiation of bislawsone from 2-hydroxy-1,4-naphthoquinone (lawsone)

**Fig. 1:**
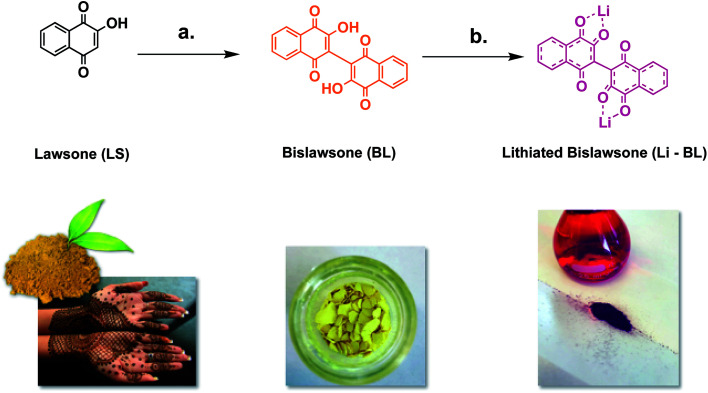
The synthesis of (a) bislawsone (BL) from lawsone (LS) using ammonium peroxodisulfate in H_2_O/MeCN (1 : 1 v/v). Reaction was kept for 3 h at 80 °C under reflux; and (b) lithiated bislawsone by treating BL with Li metal in anhydrous methanol. A color change is observed from orange (LS) to yellow (BL) to dark red (Li-BL) upon dimerization and lithiation respectively.

Bislawsone (BL) was synthesized from lawsone (LS) (Tokyo Chemical Industry >98.0%) *via* reaction with ammonium peroxodisulfate (Tokyo Chemical Industry >99.0%) according to procedures described by Inagaki *et al.* (Fig. 1).^23^ 100 mg (0.289 mmol) of BL was then dissolved in 10 mL of anhydrous methanol (Acros Organics 99.8%). 12 mg (1.73 mmol) of Li metal (Sigma Aldrich 99%), preserved in mineral oil (Spectrum Chemical), was then briefly rinsed with a few drops of diethyl ether (Fisher Scientific >99.0%) to remove excess oil and added to the methanol solution of BL. Upon addition of Li, the solution changed in color from bright yellow to dark red. This solution was stirred at room temperature for 20 min and then filtered under vacuum to remove any excess metal or metal oxide. The supernatant solution was then collected from the filtration flask and the solvent was removed under vacuum. The product was then re-dissolved in a very minimal amount of methanol and filtered again. This time the precipitate was washed with diethyl ether and collected to yield 57 mg (57% yield) of lithiated bislawsone (Li-BL) as a dark red solid.

### Material characterizations

^1^H NMR spectra were recorded in DMSO-d_6_ on the 300 MHz AVANCE III HD spectrometer (Bruker). ^13^C NMR spectra were recorded on the Unity Inova 500 MHz NMR spectrometer (Varian) at 125 MHz in DMSO-d_6_. UV-vis spectra were recorded on the Evolution 300 spectrometer (Thermo Scientific). FTIR Spectra were recorded on the Nicolet 380 IR spectrometer (Thermo Scientific) using the Attenuated Total Reflectance (ATR) technique with a diamond crystal. Tandem mass spectrometry was performed on a 4000 QTRAP (Applied Biosystems) LC-MS/MS system with an electrospray ion source. UV/vis titration studies were carried out by preparing a 3 mL solution of BL (1 × 10^−4^ M), which was titrated with lithium acetate dihydrate (LiOAc) (Acros Organics 98%) solution (3.0 × 10^−2^ M), such that a 10 μL aliquot of the LiOAc solution corresponded to 1 eq. of Li. LiOAc was chosen for all Li titration studies due to its solubility in the reaction solvent, thus providing the capability to dispense accurate volumes of the Li aliquots in solution.

### Electrochemical characterizations

Solubility tests were conducted by dissolving a few milligrams of Li-BL in *N*-methyl-2-pyrrolidone (Sigma Aldrich), de-ionized water, a mixture of ethylene carbonate (Sigma Aldrich): dimethyl carbonate (Sigma Aldrich) 1 : 1 v/v, dioxolane (Sigma Aldrich), dimethoxymethane (Sigma Aldrich) and tetraethylene glycol dimethyl ether (Sigma Aldrich). Electrochemical measurements were conducted using CR2032-type coin cells (MTI) with quartz membrane (Whatman) as a separator. The electrolyte was prepared by mixing bis(trifluoromethylsulfonyl) amine lithium salt (Sigma Aldrich) in tetraethylene glycol dimethyl ether (TEGDME). Fabrication of coin cells was conducted in an argon-filled glove box. Cyclic voltammetry and electrochemical impedance spectroscopy were measured with an Autolab PGSTAT 302 N potentiostat. Galvanostatic charge–discharge tests were performed on LAND CT2001A battery tester and on an Arbin Instruments BT-2000 battery cycler.

## Results and discussion

### NMR characterization of Li coordination to bislawsone

**Fig. 2:**
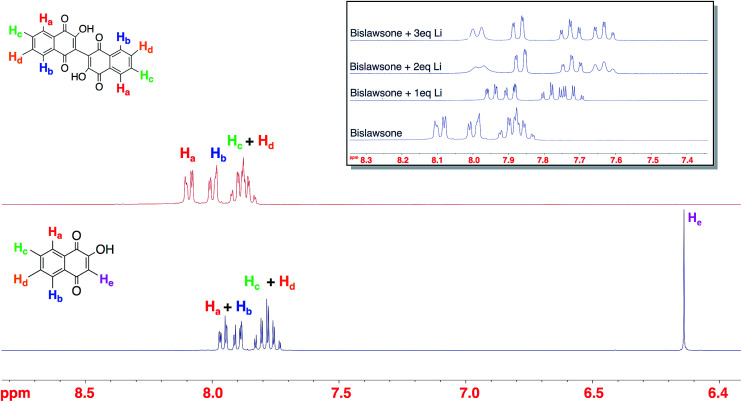
The ^1^H NMR spectra of lawsone (bottom), bislawsone (top), and lithiated bislawsone upon the addition of 1–3 eq. of Li (inset). The successful synthesis of bislawsone is characterized by the disappearance of the vinyl proton H_e_ of LS upon dimerization. A gradual shift can be observed in the aromatic protons of BL upon lithium coordination with 1 and 2 equivalents of Li indicating the binding of two lithium atoms.

The ^1^H NMR characterization of LS, BL, and Li-BL is summarized in Fig. 2. The stoichiometry of lithium coordination to BL was elucidated by monitoring the shift of aromatic protons in BL samples reacted with an increasing number of Li equivalents ranging from 1–3 eq. (Fig. 2 inset). Upon the addition of 2 eq. of Li, the doublets at *δ* 8.10 ppm and *δ* 8.00 ppm corresponding to H_a_ and H_b_ in BL exhibit significant upfield shifts to *δ* 7.99 ppm and *δ* 7.87 ppm respectively. Likewise, the multiplet at *δ* 7.88 ppm is resolved into two upfield triplets at *δ* 7.72 ppm and *δ* 7.62 ppm corresponding to H_c_ and H_d_ respectively. Further splitting can be observed for each peak due to long distance coupling that is typical of aromatic protons. No further shift is observed after the addition of 2 eq. of Li. A comparison of Li-BL that was lithiated with 2 eq. of Li and Li-BL that was lithiated with an excess of Li metal, yielded identical spectra (Fig. S3†), leading to the conclusion that Li-BL coordinates two Li atoms.

The ^13^C NMR spectra of BL displayed carbonyl peaks at 180.91 ppm and 182.30 ppm corresponding to C_1_ and C_4_ carbons, as well as a peak at 156.41 ppm corresponding to the C_2_ carbon of the hydroxyl group (Fig. S5†). Upon lithium coordination, the most dramatic shifts in the ^13^C spectrum of Li–BL (Fig. S6†) were observed for the C_1_ and C_2_ position peaks, which shifted downfield to *δ* 188.94 ppm and *δ* 167.77 ppm respectively. This observation provided supporting evidence to indicate lithium is coordinating to the carbonyl at C_1_ and the hydroxyl group at C_2_ as indicated in Fig. 1. The binding of the highly electronegative Li atom imposes a de-shielding effect upon C_1_ and C_2,_ shifting their signals downfield by a magnitude of 6.64 ppm and 11.36 ppm respectively. The remaining carbonyl C_4_ shifted by a magnitude of <*δ* 1 ppm, while no other carbon signal in the spectrum shifts more than *δ* 1–2 ppm. Since BL is a dimer of the LS molecule, the carbon atoms on each naphthoquinone ring of the dimer are identical, and thus 10 carbon peaks are displayed in the ^13^C NMR spectrum as would be in the monomer (comparison of Fig. S4 and S5†). However, since no new signals appear in the spectrum of Li-BL upon lithiation, we can conclude that the carbon atoms remain equivalent, and that Li binds to each pair of carbonyl and hydroxyl groups at the 1,2 positions of both rings.

### UV/vis and FTIR characterization of BL and Li-BL

**Fig. 3:**
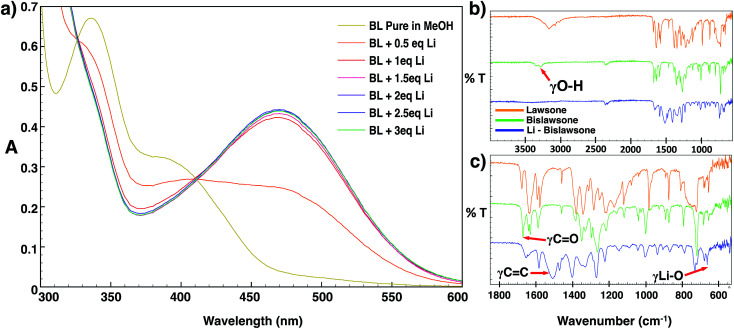
(a) The UV/vis absorption spectrum of a 1 × 10^−4^ M bislawsone solution in methanol subjected to the gradual addition of 1–3 eq. of LiOAc as the Li source. No significant shift in the spectrum is observed after the addition of 2 eq. of Li. The FTIR spectrum of lawsone (top, orange), bislawsone (middle, green) and lithiated bislawsone (bottom, blue) is shown in the regions of (b) 4000–550 cm^−1^ and (c) 1800–600 cm^−1^.

The mechanism and stoichiometry of lithium coordination to BL was further elucidated through detailed UV/vis titration studies and FTIR spectroscopy (Fig. 3a). The addition of the LiOAc solution immediately resulted in the decease of intensity of bands at 338 nm and 376 nm in the absorption spectrum of BL. A red shift of the 376 nm band towards 470 nm was further observed, and this band increased its intensity with the addition of further aliquots of Li. No further change in the intensity of the 470 nm absorption band was observed after the addition of 2 equivalents of Li. The observed red shift in the absorption spectrum of BL upon the addition of LiOAc can likely be explained by the breaking of intramolecular hydrogen bonding in BL allowing for delocalization of the molecule by isomerization to the seminaphthoquinone and catechol forms.^25,33^ Upon cell discharge of BL-based cathodes, the conjugation of the molecule may further be extended as a result of electron transfer upon lithium coordination.

The FTIR spectra of BL features a broad band at 3300–3400 cm^−1^ corresponding to the OH stretching frequency of the phenolic hydroxyl group (Fig. 3b). Additionally there are two bands of strong intensity at 1670 cm^−1^ and 1630 cm^−1^ corresponding to the stretching frequencies of the free and chelated (to the 2-hydroxyl hydrogen) carbonyl groups respectively. The band at 1630 cm^−1^ shows additional splitting corresponding to the delocalized interaction of the carbonyl with the adjacent double bond between the 2 and 3 positions of the naphthoquinone ring.^34^ Additionally, there is an intense band at 1583 cm^−1^ corresponding to the aromatic C=C stretching frequency, and a band at 1219 cm^−1^ corresponding to the C–O stretching frequency of the 2-hydroxyl group.^25^ Upon lithiation, the spectrum of Li-BL displays a broadening and decreasing intensity of the carbonyl stretching frequencies, and a new broad band of great intensity emerges in the aromatic region between 1450 to 1550 cm^−1^. The former observation can be attributed to the enhanced delocalization of the molecule that occurs upon Li chelation to the carbonyl groups, and results in the averaging of the carbon–oxygen double bond character.^25,35^ The latter can be attributed to the strengthening of the aromatic character of the naphthoquinone ring as conjugation is extended upon lithium coordination (depicted in Fig. 1).^25,34–36^ In addition, the aforementioned hydroxyl group frequency at ∼3300 cm^−1^ was found to completely disappear as the H atom of the hydroxyl group is replaced by Li; this is accompanied by the appearance of a new sharp peak at 663 cm^−1^ corresponding to the stretching frequency of the Li–O chelate (Fig. 3c).^34,35^

### Electrochemical characterizations

**Fig. 4:**
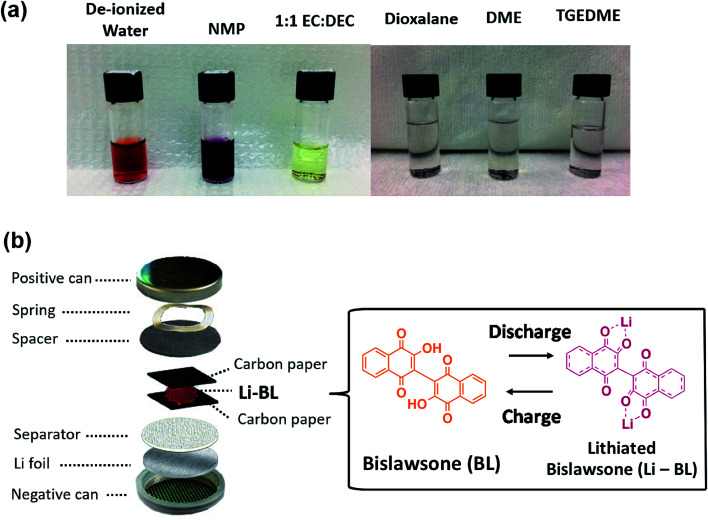
Electrochemical characterization methods for Li-BL. (a) Solubility tests of Li-BL molecule in commonly used solvents for electrode fabrication (de-ionized water and NMP) and for electrolytes ethylene carbonate (EC): dimethyl carbonate (DMC) 1 : 1 v/v, dioxolane, dimethoxymethane (DME) and tetraethylene glycol dimethyl ether (TEGDME). (b) A schematic representing the half-cell tests in coin cells used to conduct electrochemical tests on Li-BL electrodes. Li-BL electrodes were fabricated by sandwiching in between two carbon papers. This fabrication process prevents Li-BL from being dissolved in solvents commonly used during electrode fabrication such as *N*-methyl-2-pyrrolidone (NMP) and water.

The major challenge of organic molecules to be deployed in Li-ion battery chemistry is their instability in solvents during electrode fabrication and electrochemical cycling. Generally, organic electrodes with conventional Li-ion battery electrolytes exhibit catastrophic capacity fading upon initial charge–discharge cycles resulting from dissolution of electrochemically active organic species. Prior to conducting electrochemical experiments on the Li-BL molecule, we have conducted solubility tests in various solvents to establish the least soluble electrolyte system. Among the studied solvents, tetraethylene glycol dimethyl ether (TEGDME) displayed relative stability against the BL molecule as shown in Fig. 4a. Hence, the electrochemical properties of Li-BL were evaluated in CR2032 coin cells using 1 M bis(trifluoromethylsulfonyl) amine lithium salt (LiTFSI) in TEGDME as an electrolyte solution and Li foil as counter/reference electrodes. Due to solubility issues with water and *N*-methyl-2-pyrrolidone (NMP) solvent, the electrodes were fabricated using 3 : 2 wt/wt of Li-BL and conductive carbon black by mixing uniformly and being sandwiched in between two carbon papers as shown in Fig. 4b.

**Fig. 5:**
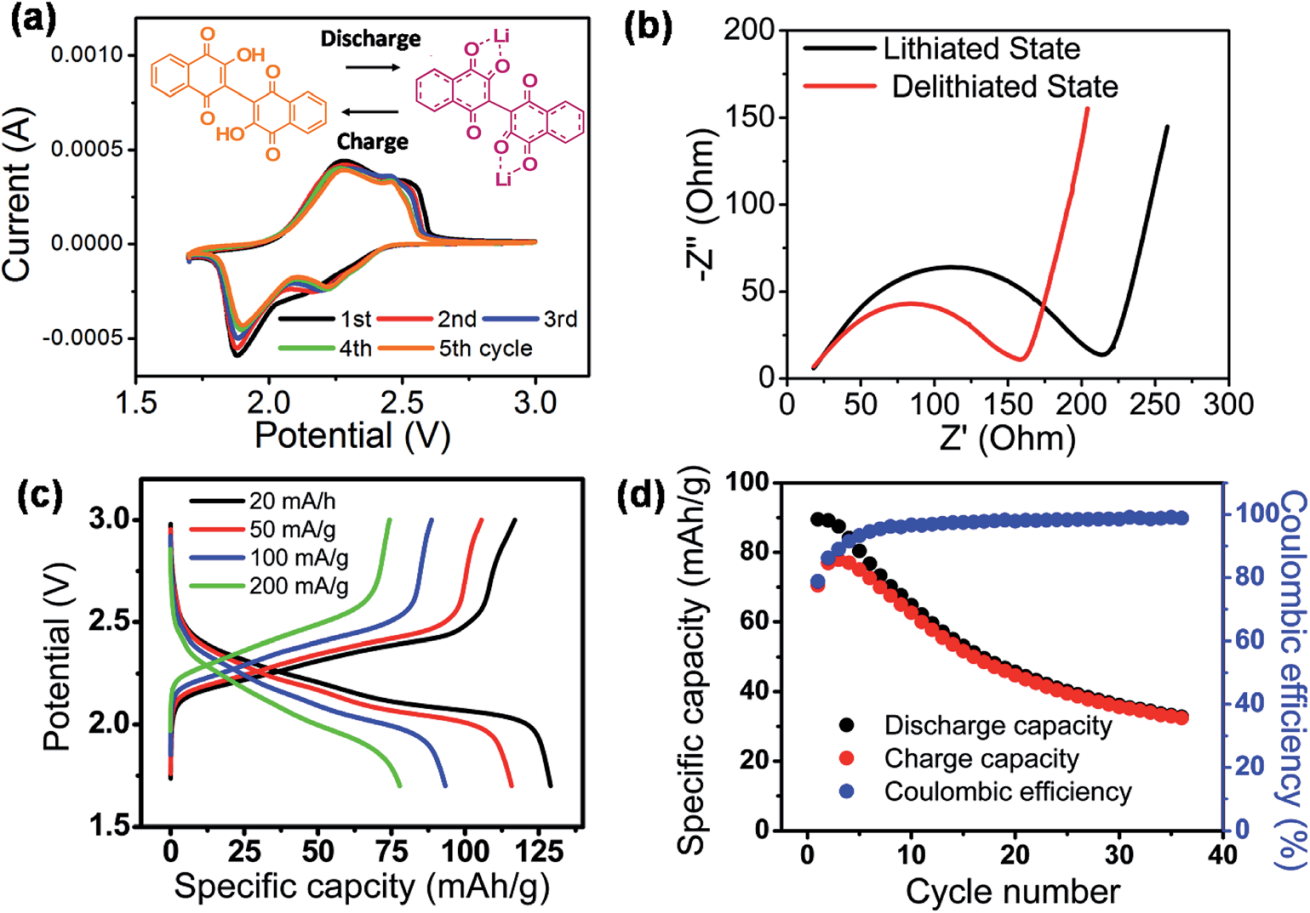
Electrochemical characterizations of Li-BL. (a) CV tests scanned between 1.7 and 3.0 V *vs.* Li/Li^+^ at rate of 0.2 mV s^−1^. (b) Electrochemical impedance spectroscopy taken at lithiated (discharged) and de-lithiated (charged) states. (c) Voltage profile of galvanostatic charge–discharge tests taken at various current densities. The presence of voltage plateau between 2.0–2.5 V *vs.* Li/Li^+^ manifests the capability of Li-BL to be used as cathode materials for Li-ion batteries. (d) Capacity retention of Li-BL electrode over repeated galvanostatic charge–discharge cycles between 0.7–3.0 V *vs.* Li/Li^+^ at current density of 50 mA g^−1^.

The fundamental electrochemical activity of Li-BL was studied with cyclic voltammetry (CV) recorded between 1.7 to 3.0 V *vs.* Li/Li^+^ at a scan rate of 0.2 mV s^−1^ (Fig. 5a). During the first cycle, a reduction peak was observed at 1.88 V followed by two broad oxidation peaks at 2.3 and 2.5 V. From the second cycle, an additional reduction peak at 2.2 V emerges along with the previously observed reduction and oxidation peaks. The first cycle can be regarded as an electrochemical activation process that has been previously reported for other organic battery materials.^37^ After the first cycle, the two pairs of redox peaks continuously appear in the subsequence cycles inferring the reversible two electron and Li-ion transfer processes. More importantly, the relatively high redox potential suggests the feasibility of Li-BL to be used as cathode materials for Li-ion batteries.^14,38^Fig. 5b shows Nyquist plots taken with electrochemical impedance spectroscopy at the lithiated (discharged) and de-lithiated (charged) state of Li-BL after 10 cycles of CV scans. It is clear that the charge-transfer resistance, a diameter of the semi-circle at high frequency region, is significantly lower at the lithiated state than that at the de-lithiated state of Li-BL. Fig. 5c represents galvanostatic charge–discharge behavior of Li-BL electrode at different current densities in TEGDME electrolyte. The charging and discharging voltage plateaus were observed between 2.1–2.5 V with insignificant polarization. The slanted voltage plateau observed in Fig. 5c is unique to carbonyl based electroactive organic molecules in which Li binding processes to various carbonyl environments in the molecules take place at slightly different potential due to the difference in LUMO energy before and after the Li binding process.^39^ Li-BL organic electrodes exhibit specific discharge capacity of 130 mA h g^−1^ at current density of 20 mA g^−1^, which corresponds to 1.7 Li per unit formula according to the following Faraday's law:
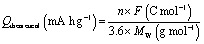
where *Q*_theoretical_ is theoretical capacity (mA h g^−1^), *n* is the number of electrons transferred, *F* is Faraday constant (96 484 C mol^−1^), and *M*_W_ is the molecular weight of active material. More notably, 61% of the discharge capacity observed at low current density (20 mA g^−1^) was retrieved at higher current density (200 mA g^−1^) indicating the fast charging and discharging capability of Li-BL electrode. The electrochemical properties of lawsone, a monomer of bislawsone, were also investigated to address the advantages of the dimerization process (Fig. S7†). A comparison of galvanostatic charge–discharge tests clearly represents that capacity fading is suppressed for Li-BL compared to the lawsone molecule. Li-BL electrodes exhibits moderate cycle performance at 50 mA g^−1^ for 35 cycles (Fig. 5d). The capacity fade observed could be attributed to the slow dissolution of Li-BL in the electrolyte under the influence of electric field.^39^ Such dissolution of Li-BL can be further suppressed by grafting onto nanocarbon materials,^40–42^ inorganic materials,^43,44^ or polymers.^45^

## Conclusions

In conclusion, we have determined that BL binds two lithium atoms, which form a coordination sphere between the 1-carbonyl and the 2-hydroxyl group oxygen atoms at each naphthoquinone ring of the dimer. Electrochemical characterizations based on Faraday's law indicated the binding of 1.7 Li atoms to BL, which was in good agreement with the data obtained from spectroscopic and titration characterizations. The Li-BL based electrodes achieved high specific capacities (130 mA h g^−1^ at 20 mA g^−1^ currents) and voltages (2.1–2.5 V) in TEGDME electrolyte, which are comparable to modern day LIB cathodes. However, although the raw material displayed stability in our electrolyte of choice, cells experienced only moderate capacity retention over 35 charge–discharge cycles. This solubility based capacity fading can be circumvented by engineering the architecture of active materials using methods such as grafting Li-BL onto graphene, polymers *etc.* Here, we have revealed the latent capability of Li-BL molecules and its use in cathode materials for Li-ion batteries. Ultimately, the cathode material reported herein is synthesized using a green chemistry approach and precludes the use of toxic transition metal compounds while minimizing harsh reaction conditions. The electrochemical performance displayed qualifies bislawsone as a cost-efficient and environmentally friendly alternative to currently studied organic and inorganic intercalation compounds. Hereby, this research has taken steps towards the realization of a sustainable future in energy storage.

## Conflicts of interest

There are no conflicts to declare.

## Supplementary Material

RA-008-C7RA12357D-s001
