# Supplementary material for: A common tattoo chemical for energy storage: henna plant-derived naphthoquinone dimer as a green and sustainable cathode material for Li-ion batteries
Source: RSC Adv. 2018 Jan 4;8(3):1576–82. doi: 10.1039/c7ra12357d (PMC9077053; doi:10.1039/c7ra12357d)
Supplement: RA-008-C7RA12357D-s001 [file RA-008-C7RA12357D-s001.pdf]

Electronic Supplementary Material (ESI) for RSC Advances.
This journal is © The Royal Society of Chemistry 2017

Electronic Supplementary Information (ESI)

A Common Tattoo Chemical for Energy Storage: Henna Plant-Derived Napthoquinone Dimer as a Green and Sustainable Cathode Material for Li-Ion Batteries

Mikhail Miroshnikov,[a][b] Keiko Kato,[c] Gangula Babu,[c] Kizmuri P. Divya,[a] Leela Mohana Reddy Arava,*[d] Pulickel M. Ajayan,*[c] and George John*[a][b]

[a]Department of Chemistry and Center for Discovery and Innovation, The City College of New York, 85 St. Nicholas Terrace, New York, NY 10031, USA. [b]Ph.D. Program in Chemistry, The Graduate Center of The City University of New York , 365 5th Ave, New York, NY, 10016, USA. [c]Department of Materials Science and Nano Engineering, Rice University, 6100 Main Street MS 325, Houston, TX 77005, USA. [d]Department of Mechanical Engineering, Wayne State University, 5050 Anthony Wayne Dr., Detroit, MI 48202, USA.

Corresponding Authors: George John, gjohn@ccny.cuny.edu; P. Ajayan, ajayan@rice.edu; Leela Mohana Reddy Arava, leela.arava@wayne.edu

Material Characterization:

Lawsone (LS):

^{1}H NMR [300 MHz, DMSO-d6, δ]: 11.63 (1H, br s) 8.00 (1H, d), 7.94 (1H, d), 7.82 (2H, m). ^{13}C NMR (125 MHz, δ]: 182.30, 180.19, 156.41, 134.96, 133.56, 132.06, 130.18, 126.13, 125.98, 115.50. FTIR (ATR, cm^{-1}): 3300, 1670, 1630, 1583, 1219; MS (ESI+) *m/z*: 197.2 (100%. M + Na) 175 (57.8, M + H).

Bislawsone (BL):

^{1}H NMR [300 MHz, DMSO-d6, δ]: 11.37 (2H, br s) 8.10 (2H, d), 8.01 (2H, d), 7.86 (4H, m). ^{13}C NMR (125 MHz, δ]: 182.30, 180.19, 156.41, 134.96, 133.56, 132.06, 130.18, 126.13, 125.98,

115.50. FTIR (ATR, cm^{-1}): 3300, 1670, 1630, 1583, 1219. MS (ESI–) *m/z*: 345.2 (100%, M – H) 346.2 (70.9, M).

Lithiated Bislawsone (Li-BL):

^{1}H NMR [300 MHz, DMSO-d6, δ]: 7.99 (1H, d), 7.87 (1H, d), 7.72 (1H, t), 7.62 (1H, t). ^{13}C NMR (125 MHz, δ]: 188.94, 181.28, 167.77, 136.18, 134.19, 131.47, 131.11, 125.86, 125.33, 117.10. FTIR (ATR, cm^{-1}): 1660, 1583, 1510, 1219, 663.

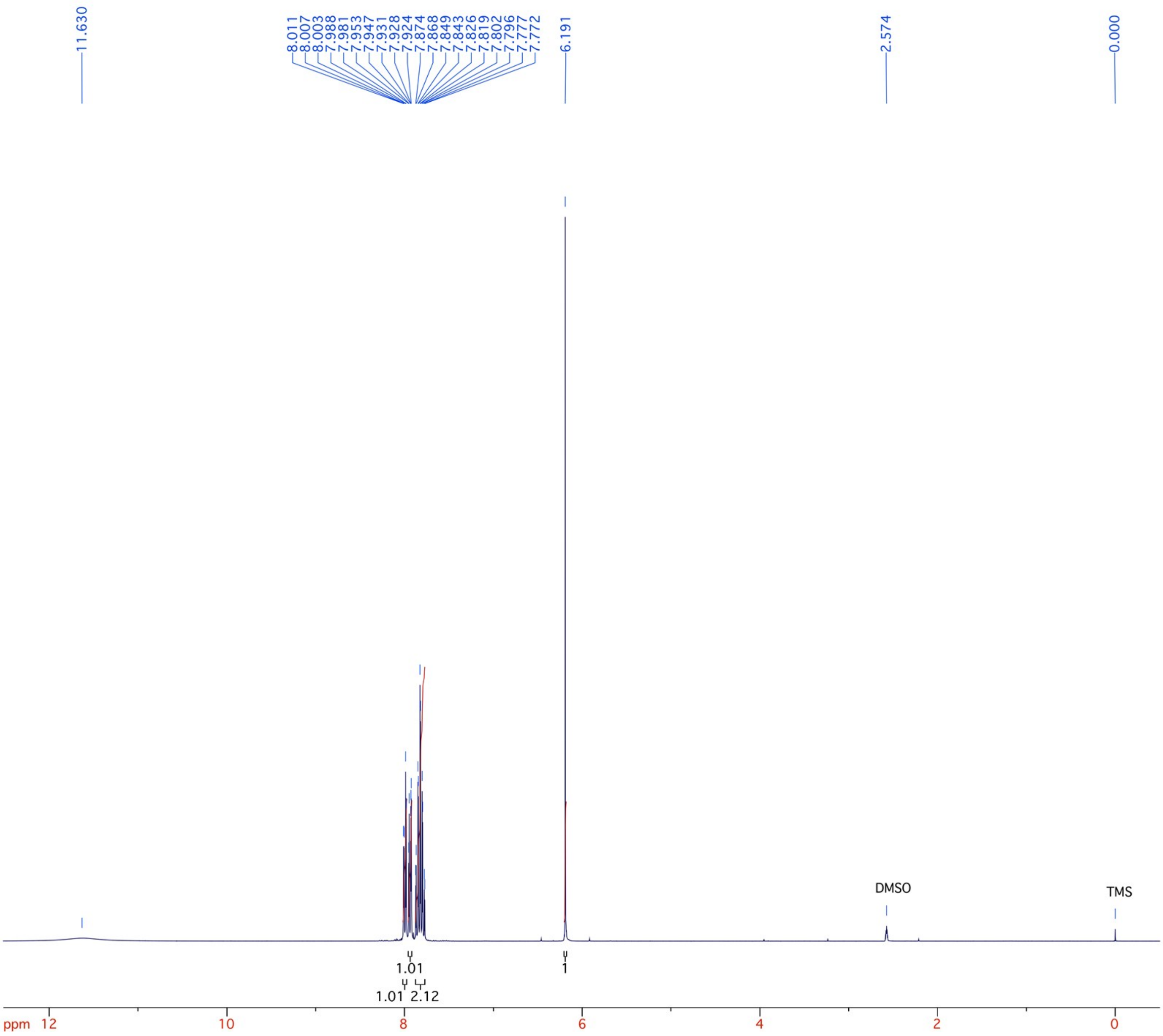

Figure S1: Expanded ^{1}H NMR spectrum of pure lawsone (LS) in DMSO with TMS at 0.00 ppm as a reference.

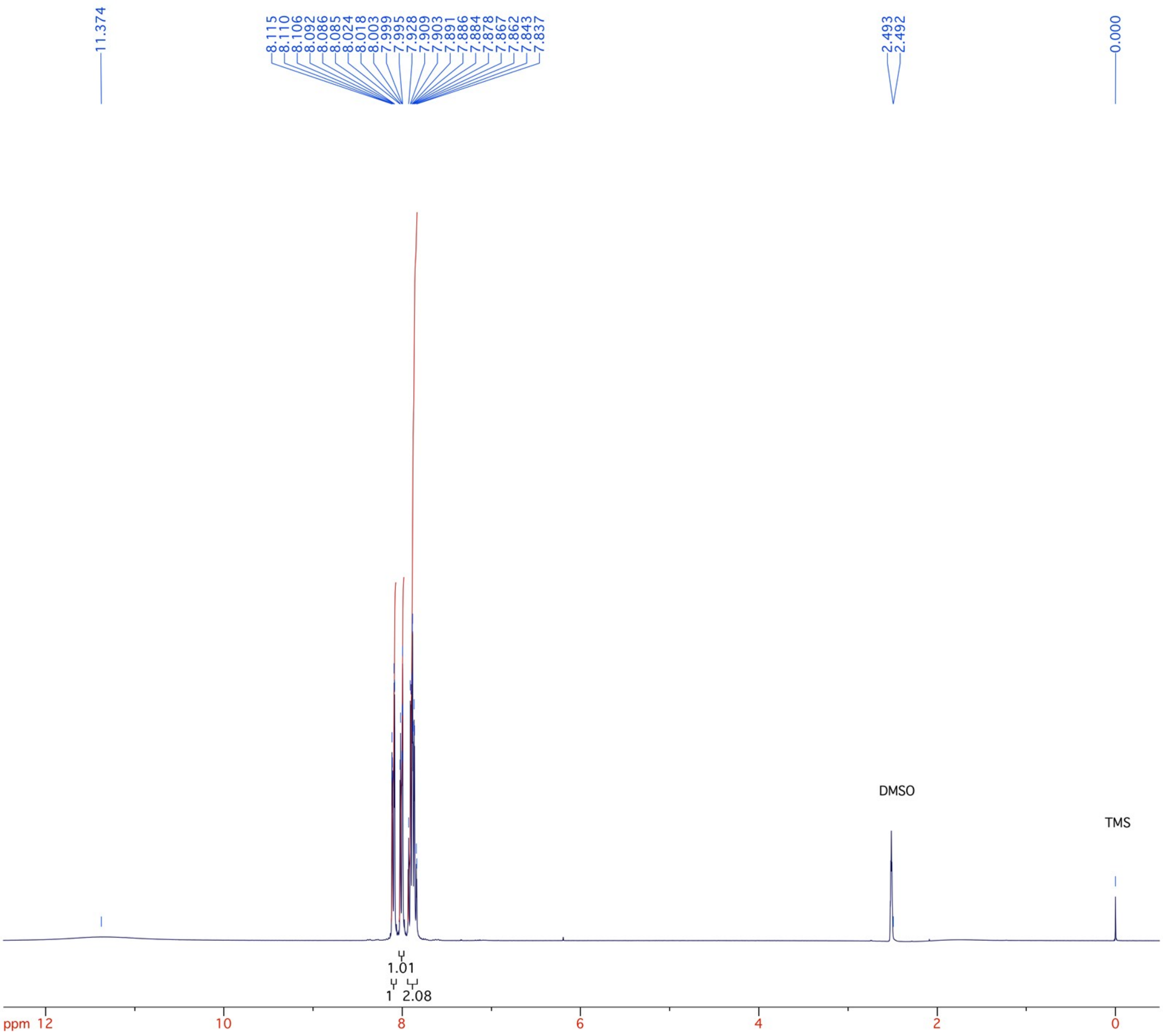

Figure S2: Expanded ^{1}H NMR spectrum of pure bislawsone (BL) in DMSO with TMS at 0.00 ppm as a reference.

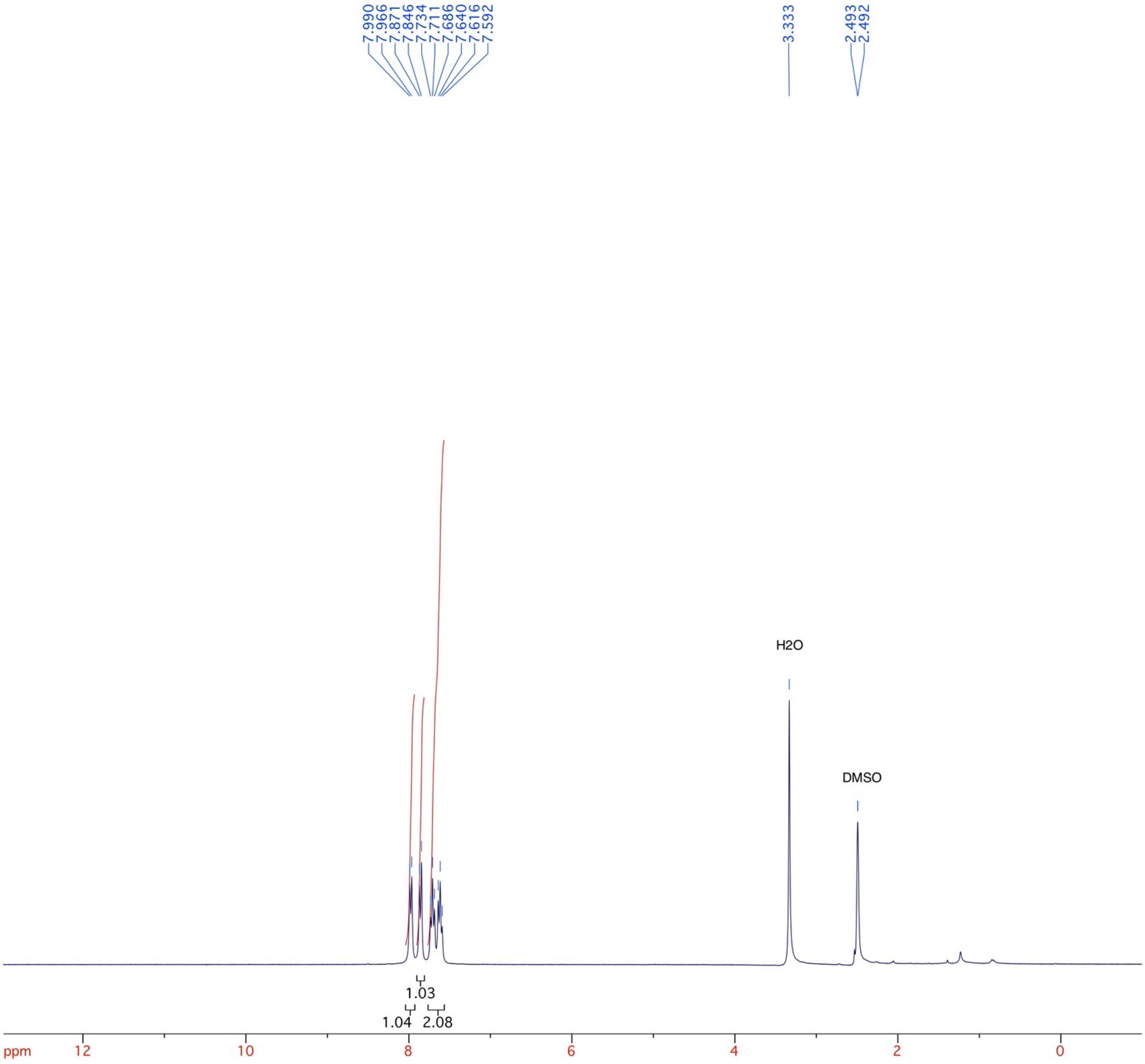

Figure S3: Expanded ^{1}H NMR spectrum of fully lithiated bislawsone (Li-BL) in DMSO with Li metal as lithium source.

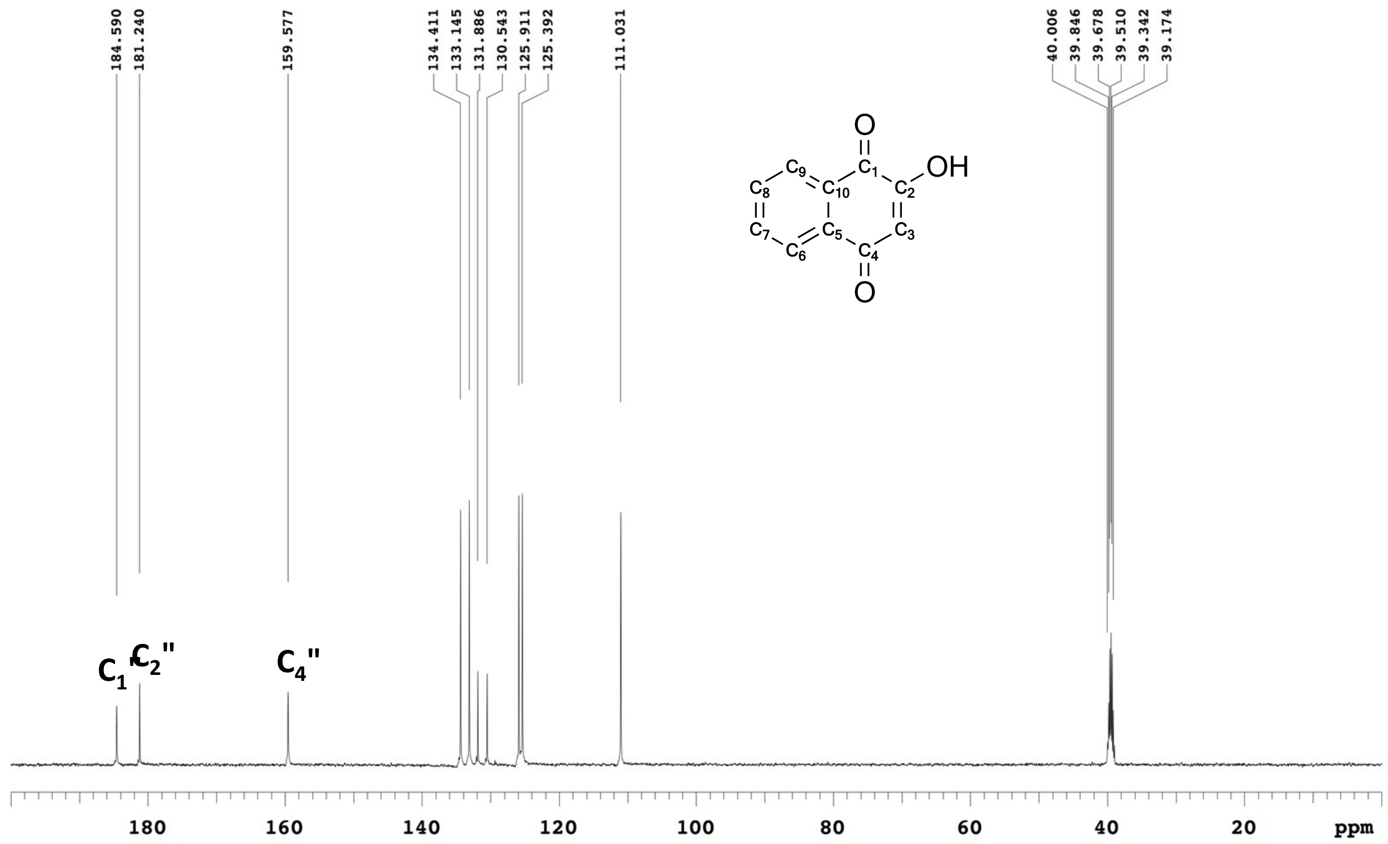

Figure S4: Annotated ^{13}C NMR spectrum of lawsone (LS) in DMSO. Carbons 1 and 4 (carbonyl), as well as carbon 2 (hydroxyl), have been labeled to highlight the significant downfield chemical shift upon subsequent dimerization to bislawsone (BL) and lithium coordination to form lithiated bislawsone (Li – BL).

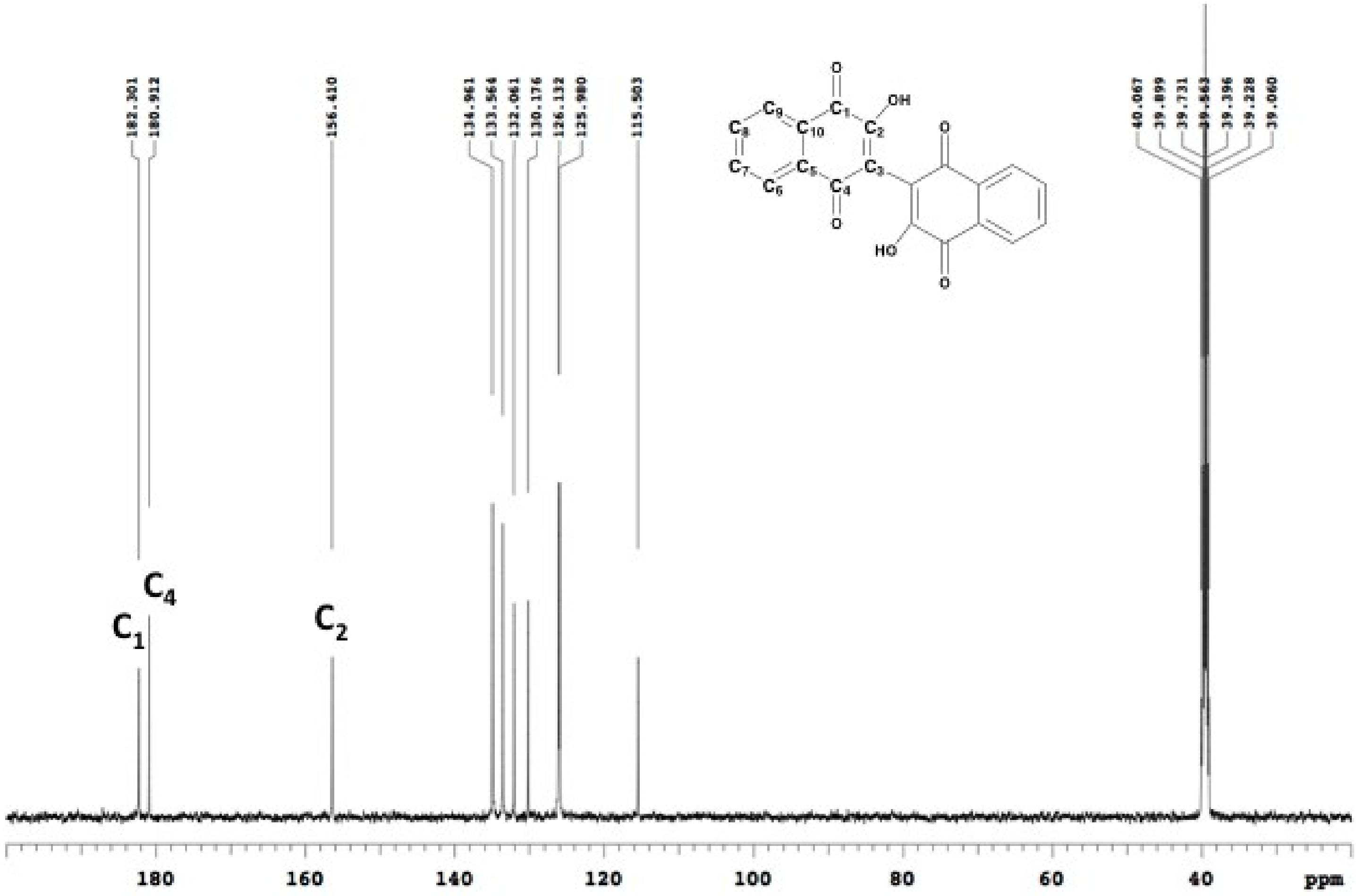

Figure S5: Annotated ^{13}C NMR spectrum of bislawsone (BL) in DMSO. Carbons 1 and 4 (carbonyl), as well as carbon 2 (hydroxyl), have been labeled to highlight the significant downfield chemical shift upon lithium coordination to form lithiated bislawsone (Li – BL).

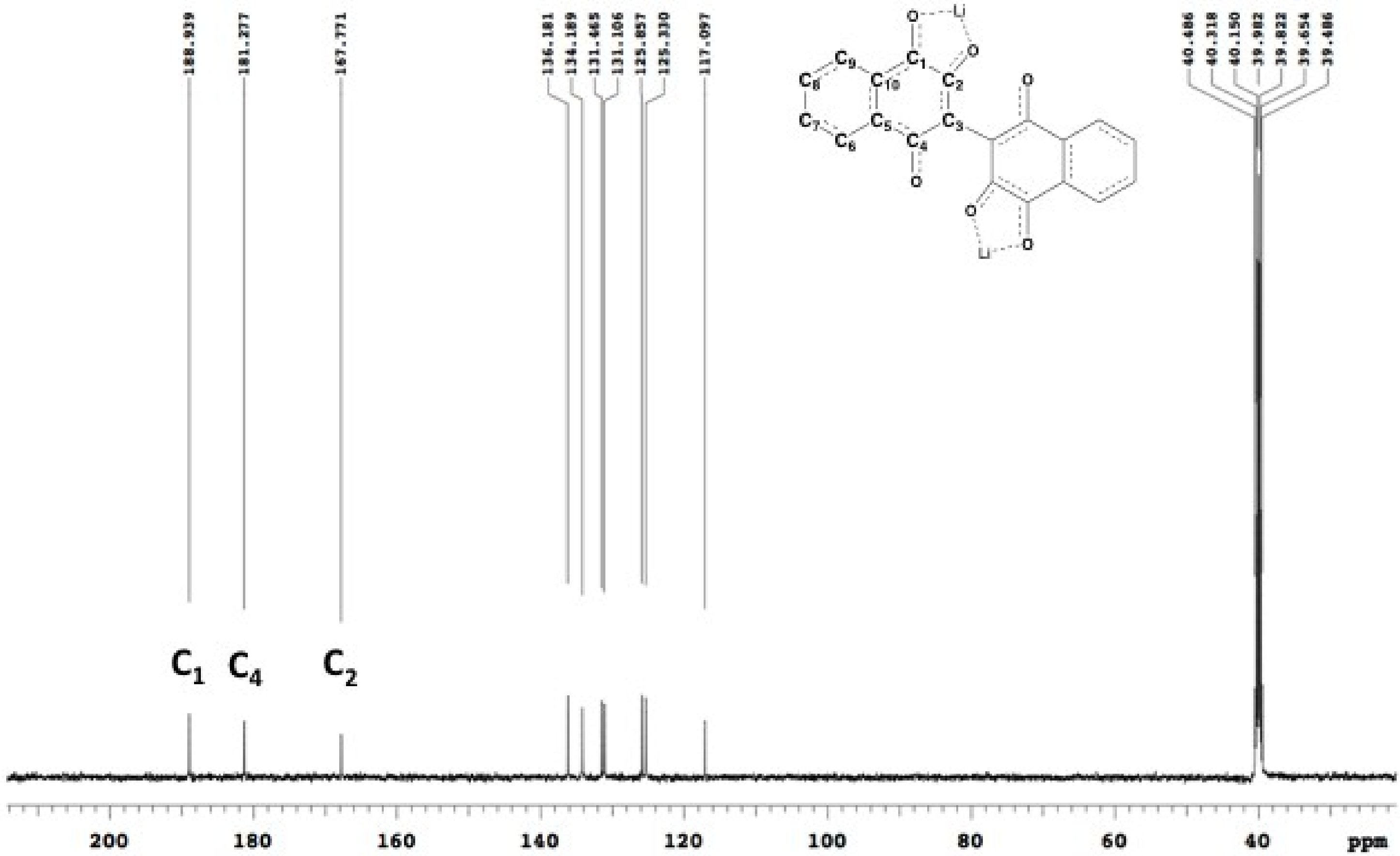

Figure S6: Annotated ^{13}C NMR spectrum of Li-BL in DMSO. Carbons 1 and 4 (carbonyl), as well as carbon 2 (hydroxyl), have been labeled to highlight the chemical shift of peaks upon lithium coordination compared to bislawsone (BL).

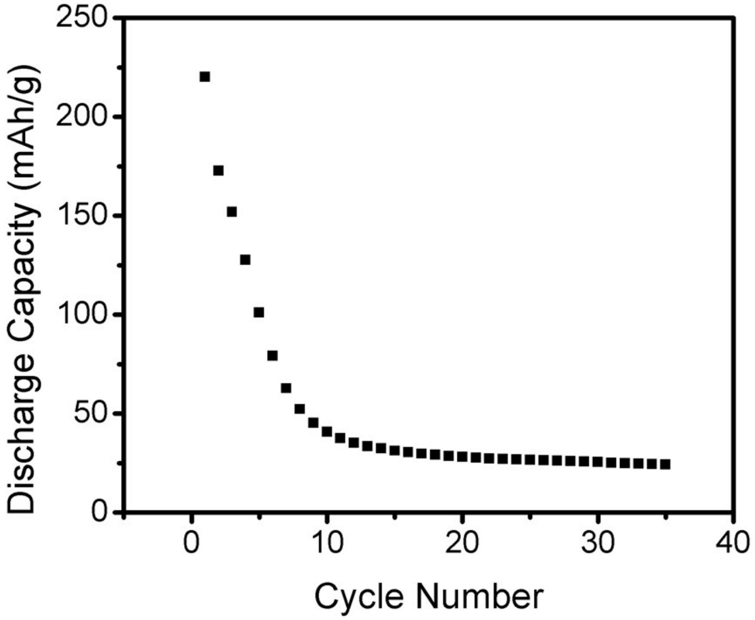

Fig S7: Capacity retention of lawsone molecule tested at current density of 50 mA/g.

Fig S7 represents galvanostatic discharge capacity conducted at current density of 50 mA/g on lawsone molecules. Although greater initial discharge (220 mAh/g) was observed for lawsone molecule than bislawsone (130 mAh/g), more pronounced capacity fade was observed for lawsone molecule than bislawsone molecule.
